# (*S*)-Benzyl 2-amino-3-(4-hydroxy­phen­yl)propanoate

**DOI:** 10.1107/S1600536808044218

**Published:** 2009-01-10

**Authors:** Shu-Na Luo, Lu Chen, Yu-Xing Gao, Peng-Xiang Xu, Yu-Fen Zhao

**Affiliations:** a Department of Chemistry and the Key Laboratory for Chemical Biology of Fujian Province, College of Chemistry and Chemical Engineering, Xiamen University, Xiamen 361005, People’s Republic of China

## Abstract

The title compound, C_16_H_17_NO_3_, adopts a folded conformation in the crystal structure. The crystal packing is stabilized by inter­molecular O—H⋯O and N—H⋯O hydrogen-bonding inter­actions. The absolute configuration was assigned assuming that the absolute configuration of the starting material l-tyrosine was retained during the synthesis.

## Related literature

For background, see: Nakamura *et al.* (1998 ▶). For the *n*-butyl analogue, see: Qian *et al.* (2006 ▶). 
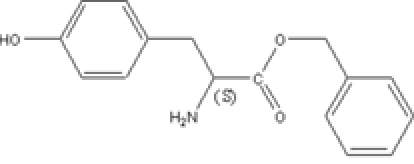


## Experimental

### 

#### Crystal data

C_16_H_17_NO_3_
*M* *_r_* = 271.31
Orthorhombic, 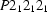

*a* = 5.1589 (2) Å
*b* = 15.1430 (4) Å
*c* = 18.6367 (6) Å
*V* = 1455.92 (8) Å^3^
*Z* = 4
Mo *K*α radiation
μ = 0.09 mm^−1^
*T* = 293 (2) K
0.25 × 0.22 × 0.18 mm

#### Data collection

Bruker SMART APEX area-detector diffractometer
Absorption correction: multi-scan (*SADABS*; Bruker, 2001 ▶) *T*_min_ = 0.979, *T*_max_ = 0.985
8138 measured reflections
1672 independent reflections
1172 reflections with *I* > 2σ(*I*)
*R*_int_ = 0.031

#### Refinement

*R*[*F* ^2^ > 2σ(*F* ^2^)] = 0.032
*wR*(*F* ^2^) = 0.076
*S* = 0.93
1672 reflections
193 parameters
H atoms treated by a mixture of independent and constrained refinement
Δρ_max_ = 0.08 e Å^−3^
Δρ_min_ = −0.10 e Å^−3^

### 

Data collection: *SMART* (Bruker, 2001 ▶); cell refinement: *SAINT* (Bruker, 2001 ▶); data reduction: *SAINT*; program(s) used to solve structure: *SHELXS97* (Sheldrick, 2008 ▶); program(s) used to refine structure: *SHELXL97* (Sheldrick, 2008 ▶); molecular graphics: *ORTEP-3 for Windows* (Farrugia, 1997 ▶); software used to prepare material for publication: *SHELXL97*.

## Supplementary Material

Crystal structure: contains datablocks I, global. DOI: 10.1107/S1600536808044218/wk2093sup1.cif

Structure factors: contains datablocks I. DOI: 10.1107/S1600536808044218/wk2093Isup2.hkl

Additional supplementary materials:  crystallographic information; 3D view; checkCIF report

## Figures and Tables

**Table 1:** Hydrogen-bond geometry (Å, °)

*D*—H⋯*A*	*D*—H	H⋯*A*	*D*⋯*A*	*D*—H⋯*A*
O3—H3⋯N1^i^	0.93 (3)	1.82 (3)	2.718 (3)	163 (2)
N1—H1*A*⋯O1^ii^	0.88 (2)	2.21 (3)	3.030 (3)	156 (2)

Symmetry codes: (i) 
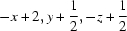
; (ii) 
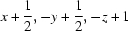
.

**Fig. 1.**
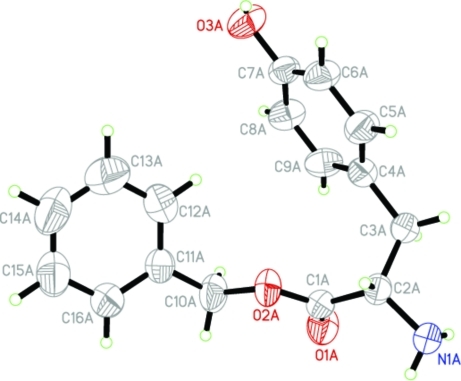
The molecular structure of (I), showing 50% probability displacement ellipsoids (arbitrary spheres for H atoms).

## supplementary crystallographic information

### Comment

The title compound, (I), is a valuable protected amino acid, which is used not only for polypeptide synthesis but also for the synthesis of (4-Boronophenyl)alanine (BPA), which is clinically used for the treatment of malignant melanoma (Nakamura *et al.*, 1998).

The molecular structure of (I) is shown in Fig. 1. The molecule adopts a somewhat folded or U-shaped conformation as evidenced in the C1/C2/C3/C4 torsion angle of 58.9 (3)°. Despite the adoption of this conformation, there is no evidence for significant intramolecular C—H···π interactions. In terms of geometric parameters and overall conformation, the structure of (I) resembles that of the *n*-butyl analogue (Qian *et al.*, 2006). Hydrogen bonding plays a significant role in stabilizing the crystal structure; see Table 1 for geometric parameters and symmetry operations. The most prominent link occurs between the phenol H and the amine N atoms, to form chains along the *c* axis. Molecules are connected into a three-dimensional array by O—H···O and N—H···O intermolecular hydrogen-bonding interactions. The absolute configuration of (I) was assigned assuming that the absolute configuration of the starting material *L*-tyrosine was retained during the synthesis.

### Experimental

To a solution of *L*-tyrosine (10 g, 55 mmol) and benzyl alcohol (25 ml) in benzene (120 ml) was added *p*-toluenesulfonic acid monohydrate (12.6 g, 66 mmol) at room temperature. The water generated in the reaction was separated by benzene azeotropic distillation for 3 h, and the white precipitate was filtered off. The filtrate was washed with sodium bicarbonate solution, dried over anhydrous magnesium sulfate, filtered and concentrated. Purification by recrystallization from ethanol gave the titled compound as a white solid (12 g). Single crystals of (I) were obtained by slow evaporation of an ethyl acetate solution.

### Refinement

The H atoms were positioned geometrically (C—H = 0.93, 0.98 or 0.97 Å for phenyl, methine or methylene H atoms, respectively) and were included in the refinement in the riding-model approximation. The isotropic displacement parameters were set at 1.2 times *U*_eq_ of the parent atoms. The NH and OH positions were located from difference Fourier maps and their positions and an isotropic displacement parameter refined. In the absence of significant anomalous scattering effects, Friedel pairs were merged.

### Crystal data

**Table:**

C_16_H_17_NO_3_	*F*(000) = 576
*M**_r_* = 271.31	*D*_x_ = 1.238 Mg m^−^^3^
Orthorhombic, *P*2_1_2_1_2_1_	Mo *K*α radiation, λ = 0.71073 Å
Hall symbol: P 2ac 2ab	Cell parameters from 2651 reflections
*a* = 5.1589 (2) Å	θ = 2.7–32.7°
*b* = 15.1430 (4) Å	µ = 0.09 mm^−^^1^
*c* = 18.6367 (6) Å	*T* = 293 K
*V* = 1455.92 (8) Å^3^	Plate, colorless
*Z* = 4	0.25 × 0.22 × 0.18 mm

### Data collection

**Table:**

Bruker APEX area-detector diffractometer	1672 independent reflections
Radiation source: fine-focus sealed tube	1172 reflections with *I* > 2σ(*I*)
graphite	*R*_int_ = 0.031
φ and ω scans	θ_max_ = 26.0°, θ_min_ = 2.7°
Absorption correction: multi-scan (*SADABS*; Bruker, 2001)	*h* = −5→6
*T*_min_ = 0.979, *T*_max_ = 0.985	*k* = −18→16
8138 measured reflections	*l* = −22→22

### Refinement

**Table:**

Refinement on *F*^2^	Primary atom site location: structure-invariant direct methods
Least-squares matrix: full	Secondary atom site location: difference Fourier map
*R*[*F*^2^ > 2σ(*F*^2^)] = 0.032	Hydrogen site location: geom CH, N and OH from difmap
*wR*(*F*^2^) = 0.076	H atoms treated by a mixture of independent and constrained refinement
*S* = 0.93	*w* = 1/[σ^2^(*F*_o_^2^) + (0.0454*P*)^2^] where *P* = (*F*_o_^2^ + 2*F*_c_^2^)/3
1672 reflections	(Δ/σ)_max_ < 0.001
193 parameters	Δρ_max_ = 0.08 e Å^−^^3^
0 restraints	Δρ_min_ = −0.10 e Å^−^^3^

### Special details

**Table:**

Geometry. All e.s.d.'s (except the e.s.d. in the dihedral angle between two l.s. planes) are estimated using the full covariance matrix. The cell e.s.d.'s are taken into account individually in the estimation of e.s.d.'s in distances, angles and torsion angles; correlations between e.s.d.'s in cell parameters are only used when they are defined by crystal symmetry. An approximate (isotropic) treatment of cell e.s.d.'s is used for estimating e.s.d.'s involving l.s. planes.
Refinement. Refinement of *F*^2^ against ALL reflections. The weighted *R*-factor *wR* and goodness of fit *S* are based on *F*^2^, conventional *R*-factors *R* are based on *F*, with *F* set to zero for negative *F*^2^. The threshold expression of *F*^2^ > σ(*F*^2^) is used only for calculating *R*-factors(gt) *etc*. and is not relevant to the choice of reflections for refinement. *R*-factors based on *F*^2^ are statistically about twice as large as those based on *F*, and *R*- factors based on ALL data will be even larger.

### Fractional atomic coordinates and isotropic or equivalent isotropic displacement parameters (Å^2^)

**Table:**

	*x*	*y*	*z*	*U*_iso_*/*U*_eq_	
O1	0.4947 (3)	0.30756 (10)	0.45476 (9)	0.0650 (5)	
O2	0.8528 (3)	0.38687 (8)	0.43244 (8)	0.0538 (4)	
O3	0.7826 (4)	0.60066 (9)	0.17256 (9)	0.0648 (5)	
N1	0.7847 (5)	0.15910 (11)	0.39585 (11)	0.0499 (5)	
C1	0.7006 (4)	0.31643 (12)	0.42536 (11)	0.0451 (5)	
C2	0.8176 (4)	0.25132 (12)	0.37341 (10)	0.0441 (5)	
H2A	1.0032	0.2638	0.3688	0.053*	
C3	0.6894 (5)	0.26200 (12)	0.29951 (11)	0.0564 (6)	
H3B	0.7642	0.2188	0.2672	0.068*	
H3C	0.5065	0.2484	0.3042	0.068*	
C4	0.7151 (5)	0.35192 (13)	0.26566 (11)	0.0491 (5)	
C5	0.9175 (5)	0.37134 (14)	0.21981 (12)	0.0598 (6)	
H5A	1.0392	0.3278	0.2096	0.072*	
C6	0.9440 (5)	0.45339 (13)	0.18877 (12)	0.0558 (6)	
H6A	1.0822	0.4644	0.1580	0.067*	
C7	0.7680 (4)	0.51880 (12)	0.20290 (11)	0.0470 (5)	
C8	0.5653 (5)	0.50079 (14)	0.24802 (13)	0.0591 (6)	
H8A	0.4431	0.5443	0.2578	0.071*	
C9	0.5413 (5)	0.41866 (15)	0.27908 (12)	0.0593 (6)	
H9A	0.4034	0.4081	0.3100	0.071*	
C10	0.7522 (5)	0.45997 (13)	0.47444 (13)	0.0634 (6)	
H10A	0.7322	0.4426	0.5242	0.076*	
H10B	0.5845	0.4782	0.4562	0.076*	
C11	0.9434 (5)	0.53384 (13)	0.46821 (13)	0.0531 (6)	
C12	1.0032 (7)	0.56917 (19)	0.40256 (15)	0.0943 (10)	
H12A	0.9254	0.5469	0.3613	0.113*	
C13	1.1791 (9)	0.6379 (2)	0.39742 (18)	0.1095 (12)	
H13A	1.2166	0.6621	0.3527	0.131*	
C14	1.2967 (7)	0.67010 (17)	0.4564 (2)	0.0909 (9)	
H14A	1.4155	0.7161	0.4526	0.109*	
C15	1.2409 (7)	0.63529 (17)	0.52081 (19)	0.0889 (9)	
H15A	1.3218	0.6573	0.5617	0.107*	
C16	1.0650 (6)	0.56733 (15)	0.52689 (14)	0.0687 (7)	
H16A	1.0287	0.5439	0.5719	0.082*	
H1A	0.816 (5)	0.1545 (14)	0.4419 (14)	0.060 (7)*	
H1B	0.613 (7)	0.1410 (17)	0.3878 (16)	0.091 (9)*	
H3	0.941 (6)	0.6089 (16)	0.1494 (15)	0.094 (10)*	

### Atomic displacement parameters (Å^2^)

**Table:**

	*U*^11^	*U*^22^	*U*^33^	*U*^12^	*U*^13^	*U*^23^
O1	0.0604 (10)	0.0618 (9)	0.0728 (11)	−0.0057 (8)	0.0199 (9)	−0.0112 (8)
O2	0.0563 (8)	0.0399 (7)	0.0652 (9)	−0.0023 (7)	0.0044 (8)	−0.0138 (7)
O3	0.0676 (11)	0.0502 (8)	0.0766 (11)	0.0065 (8)	0.0092 (10)	0.0187 (8)
N1	0.0652 (13)	0.0378 (9)	0.0467 (11)	−0.0001 (9)	−0.0009 (11)	0.0004 (8)
C1	0.0508 (12)	0.0408 (11)	0.0437 (11)	0.0019 (10)	−0.0031 (12)	−0.0007 (9)
C2	0.0483 (11)	0.0380 (10)	0.0460 (11)	−0.0029 (9)	0.0014 (11)	−0.0021 (9)
C3	0.0777 (15)	0.0465 (11)	0.0451 (11)	−0.0146 (11)	−0.0028 (13)	0.0018 (10)
C4	0.0586 (13)	0.0472 (11)	0.0415 (10)	−0.0079 (11)	−0.0026 (12)	−0.0003 (10)
C5	0.0699 (15)	0.0461 (12)	0.0635 (14)	0.0064 (12)	0.0128 (13)	0.0032 (11)
C6	0.0559 (13)	0.0546 (12)	0.0570 (14)	0.0007 (12)	0.0147 (12)	0.0098 (12)
C7	0.0496 (12)	0.0444 (11)	0.0471 (11)	−0.0031 (10)	−0.0049 (12)	0.0088 (10)
C8	0.0538 (14)	0.0585 (13)	0.0649 (14)	0.0130 (12)	0.0075 (14)	0.0120 (12)
C9	0.0514 (13)	0.0703 (15)	0.0563 (14)	−0.0020 (12)	0.0105 (12)	0.0134 (12)
C10	0.0730 (16)	0.0474 (11)	0.0698 (13)	0.0002 (13)	0.0112 (15)	−0.0183 (11)
C11	0.0631 (14)	0.0418 (11)	0.0544 (13)	0.0020 (11)	0.0010 (13)	−0.0077 (10)
C12	0.138 (3)	0.0831 (18)	0.0616 (17)	−0.036 (2)	−0.002 (2)	−0.0094 (15)
C13	0.163 (4)	0.088 (2)	0.0770 (19)	−0.036 (2)	0.021 (2)	0.0098 (18)
C14	0.106 (2)	0.0549 (14)	0.111 (2)	−0.0181 (16)	0.009 (2)	−0.0092 (17)
C15	0.104 (2)	0.0669 (16)	0.096 (2)	−0.0152 (18)	−0.025 (2)	−0.0109 (17)
C16	0.0863 (18)	0.0553 (14)	0.0645 (15)	−0.0028 (14)	−0.0128 (16)	−0.0006 (13)

### Geometric parameters (Å, °)

**Table:**

O1—C1	1.203 (2)	C6—H6A	0.9300
O2—C1	1.331 (2)	C7—C8	1.369 (3)
O2—C10	1.452 (2)	C8—C9	1.377 (3)
O3—C7	1.365 (2)	C8—H8A	0.9300
O3—H3	0.93 (3)	C9—H9A	0.9300
N1—C2	1.467 (3)	C10—C11	1.496 (3)
N1—H1A	0.88 (2)	C10—H10A	0.9700
N1—H1B	0.94 (3)	C10—H10B	0.9700
C1—C2	1.508 (3)	C11—C16	1.359 (3)
C2—C3	1.536 (3)	C11—C12	1.371 (4)
C2—H2A	0.9800	C12—C13	1.384 (5)
C3—C4	1.506 (3)	C12—H12A	0.9300
C3—H3B	0.9700	C13—C14	1.348 (5)
C3—H3C	0.9700	C13—H13A	0.9300
C4—C9	1.374 (3)	C14—C15	1.341 (4)
C4—C5	1.381 (3)	C14—H14A	0.9300
C5—C6	1.377 (3)	C15—C16	1.377 (4)
C5—H5A	0.9300	C15—H15A	0.9300
C6—C7	1.369 (3)	C16—H16A	0.9300
			
C1—O2—C10	116.97 (16)	C8—C7—C6	118.71 (18)
C7—O3—H3	111.3 (16)	C7—C8—C9	120.4 (2)
C2—N1—H1A	109.5 (15)	C7—C8—H8A	119.8
C2—N1—H1B	109.9 (16)	C9—C8—H8A	119.8
H1A—N1—H1B	108 (3)	C4—C9—C8	121.9 (2)
O1—C1—O2	124.43 (19)	C4—C9—H9A	119.0
O1—C1—C2	124.95 (19)	C8—C9—H9A	119.0
O2—C1—C2	110.58 (18)	O2—C10—C11	107.02 (19)
N1—C2—C1	113.15 (16)	O2—C10—H10A	110.3
N1—C2—C3	107.82 (16)	C11—C10—H10A	110.3
C1—C2—C3	109.55 (17)	O2—C10—H10B	110.3
N1—C2—H2A	108.7	C11—C10—H10B	110.3
C1—C2—H2A	108.7	H10A—C10—H10B	108.6
C3—C2—H2A	108.7	C16—C11—C12	118.0 (2)
C4—C3—C2	115.65 (16)	C16—C11—C10	121.4 (2)
C4—C3—H3B	108.4	C12—C11—C10	120.6 (2)
C2—C3—H3B	108.4	C11—C12—C13	120.2 (3)
C4—C3—H3C	108.4	C11—C12—H12A	119.9
C2—C3—H3C	108.4	C13—C12—H12A	119.9
H3B—C3—H3C	107.4	C14—C13—C12	120.7 (3)
C9—C4—C5	116.71 (19)	C14—C13—H13A	119.6
C9—C4—C3	122.1 (2)	C12—C13—H13A	119.6
C5—C4—C3	121.2 (2)	C15—C14—C13	119.4 (3)
C6—C5—C4	121.8 (2)	C15—C14—H14A	120.3
C6—C5—H5A	119.1	C13—C14—H14A	120.3
C4—C5—H5A	119.1	C14—C15—C16	120.6 (3)
C7—C6—C5	120.4 (2)	C14—C15—H15A	119.7
C7—C6—H6A	119.8	C16—C15—H15A	119.7
C5—C6—H6A	119.8	C11—C16—C15	121.1 (3)
O3—C7—C8	118.5 (2)	C11—C16—H16A	119.4
O3—C7—C6	122.7 (2)	C15—C16—H16A	119.4
			
C10—O2—C1—O1	−4.7 (3)	C6—C7—C8—C9	0.7 (3)
C10—O2—C1—C2	173.19 (16)	C5—C4—C9—C8	0.6 (3)
O1—C1—C2—N1	−41.7 (3)	C3—C4—C9—C8	−180.0 (2)
O2—C1—C2—N1	140.49 (19)	C7—C8—C9—C4	−0.8 (4)
O1—C1—C2—C3	78.7 (2)	C1—O2—C10—C11	−174.17 (19)
O2—C1—C2—C3	−99.2 (2)	O2—C10—C11—C16	−119.4 (2)
N1—C2—C3—C4	−177.03 (19)	O2—C10—C11—C12	59.8 (3)
C1—C2—C3—C4	59.4 (3)	C16—C11—C12—C13	−1.1 (4)
C2—C3—C4—C9	−87.4 (3)	C10—C11—C12—C13	179.6 (3)
C2—C3—C4—C5	92.1 (2)	C11—C12—C13—C14	1.0 (6)
C9—C4—C5—C6	−0.2 (3)	C12—C13—C14—C15	−0.3 (6)
C3—C4—C5—C6	−179.7 (2)	C13—C14—C15—C16	−0.1 (5)
C4—C5—C6—C7	0.1 (4)	C12—C11—C16—C15	0.7 (4)
C5—C6—C7—O3	−178.8 (2)	C10—C11—C16—C15	179.9 (2)
C5—C6—C7—C8	−0.3 (3)	C14—C15—C16—C11	−0.1 (4)
O3—C7—C8—C9	179.2 (2)		

### Hydrogen-bond geometry (Å, °)

**Table:**

*D*—H···*A*	*D*—H	H···*A*	*D*···*A*	*D*—H···*A*
O3—H3···N1^i^	0.93 (3)	1.82 (3)	2.718 (3)	163 (2)
N1—H1A···O1^ii^	0.88 (2)	2.21 (3)	3.030 (3)	156 (2)

Symmetry codes: (i) −*x*+2, *y*+1/2, −*z*+1/2; (ii) *x*+1/2, −*y*+1/2, −*z*+1.
